# Improving estimates of insecticide-treated mosquito net coverage from household surveys: using geographic coordinates to account for endemicity

**DOI:** 10.1186/1475-2875-13-254

**Published:** 2014-07-04

**Authors:** Clara R Burgert, Sarah EK Bradley, Fred Arnold, Erin Eckert

**Affiliations:** 1 DHS Program, ICF International, 530 Gaither Road, Suite 500, Rockville, MD 20850, USA; 2 Department of Demography, University of California, 2232 Piedmont Ave, Berkeley, CA 94720, USA; 3 President’s Malaria Initiative, US Agency for International Development, USAID/PMI, Global Health Fellows, 1201 Pennsylvania Ave. NW, Suite 315, Washington, DC 20004, USA

**Keywords:** Insecticide-treated nets (ITNs), Malaria interventions, Intervention coverage, Demographic and Health Survey (DHS), Malaria Indicator Survey (MIS), Roll Back Malaria (RBM), Global Positioning System (GPS), Geographic Information Systems (GIS), Endemicity, Malaria Atlas Project (MAP)

## Abstract

**Background:**

Coverage estimates of insecticide-treated nets (ITNs) are often calculated at the national level, but are intended to be a proxy for coverage among the population at risk of malaria. The analysis uses data for surveyed households, linking survey enumeration areas (clusters) with levels of malaria endemicity and adjusting coverage estimates based on the population at risk. This analysis proposes an approach that is not dependent on being able to identify malaria risk in a location during the survey design (since survey samples are typically selected on the basis of census sampling frames that do not include information on malaria zones), but rather being able to assign risk zones after a survey has already been completed.

**Methods:**

The analysis uses data from 20 recent nationally representative Demographic and Health Survey (DHS), Malaria Indicator Surveys (MIS), an AIDS Indicator Survey (AIS), and an Anemia and Malaria Prevalence Survey (AMP). The malaria endemicity classification was assigned from the Malaria Atlas Project (MAP) 2010 interpolated data layers, using the Geographic Positioning System (GPS) location of the survey clusters. National ITN coverage estimates were compared with coverage estimates in intermediate/high endemicity zones (i.e., the population at risk of malaria) to determine whether the difference between estimates was statistically different from zero (p-value <0.5).

**Results:**

Endemicity varies substantially in eight of the 20 studied countries. In these countries with heterogeneous transmission of malaria, stratification of households by endemicity zones shows that ITN coverage in intermediate/high endemicity zones is significantly higher than ITN coverage at the national level (Burundi, Kenya, Namibia, Rwanda, Tanzania, Senegal, Zambia, and Zimbabwe.). For example in Zimbabwe, the national ownership of ITNs is 28%, but ownership in the intermediate/high endemicity zone is 46%.

**Conclusion:**

Incorporating this study’s basic and easily reproducible approach into estimates of ITN coverage is applicable and even preferable in countries with areas at no/low risk of malaria and will help ensure that the highest-quality data are available to inform programmatic decisions in countries affected by malaria. The extension of this type of analysis to other malaria interventions can provide further valuable information to support evidence-based decision-making.

## Background

Accurate estimates of mosquito net ownership are important for measuring progress toward intervention coverage goals as programs move toward malaria eradication. The Global Malaria Action Plan (GMAP) targets for malaria prevention interventions are supposed to be restricted to the population at risk of malaria infection [1]. In this study, a methodological approach to classifying the population at risk of malaria for the calculation of intervention coverage indicators from nationally representative population-based surveys is proposed. The study examines two Roll Back Malaria (RBM) outcome indicators for population coverage estimates of ITN ownership (Table 1) based on the population at risk of malaria by taking into account the malaria risk in survey locations and geographic malaria prevalence estimates from the Malaria Atlas Project 2010 data layer [2].

**Table 1:** Outcome indicators used in this analysis

**Indicator description**	**Numerator**	**Denominator**	**Note**
Proportion of households with at least one ITN	Number of households surveyed with at least one ITN	Total number of households surveyed	Existing indicator
Proportion of households with at least one ITN for every two people	Number of households with at least one ITN for every two people	Total number of households surveyed	New indicator, June 2013

Note: These indicators were developed by the RBM Monitoring and Evaluation Reference Group Survey and Indicator Task Force [3].

Standardly, RBM and the US Government funded President’s Malaria Initiative (PMI) use population-based surveys to measure intervention coverage indicators for malaria prevention and treatment. Nationally representative population-based surveys include the Demographic and Health Surveys (DHS), the Malaria Indicator Surveys (MIS), the AIDS Indicator Surveys (AIS), and the Multiple Indicator Cluster Surveys (MICS). These surveys are not all solely malaria focused but they often include malaria-related questions allowing for the construction and calculation of malaria intervention coverage indicators. The RBM publication on population-based (household) survey indicators for malaria control states that for most sub-Saharan African countries, where malaria is endemic or epidemic prone throughout, reporting indicators at a national level with urban and rural stratification is sufficient [3]. The document further states that countries with heterogeneous endemicity, such as countries with deserts or high altitude areas, should collect information to classify survey locations (enumeration areas) by malarial zones and use this information for stratification of reporting indicators.

For surveys that are malaria focused, the Malaria Indicator Survey sampling manual gives specific recommendations on identifying malaria endemicity zones and on drawing a sample taking endemicity into account [4]. Clear guidance on how to quantify the population at risk or identify malarial zones and how to apply this information in calculating intervention coverage estimates remains a challenge in nationally representative population-based surveys that are not malaria focused. These surveys are usually designed to have representative samples for subnational administrative units, but not for specific malaria risk zones.

Different groups have developed their own approaches to classify the population at risk of malaria in for datasets or surveys that did not stratify the country by malaria zones. The World Health Organization (WHO), in the World Malaria Reports since 2010, has classified an area (second administrative or lower level) as being at high risk of malaria if there was at least one case of malaria per 1,000 population per year. All others areas are considered to be at low risk [5]. Other groups have leveraged sophisticated geo-statistical modeling techniques to create interpolated gridded surfaces estimating malaria risk. The “Mapping Malaria Risk in Africa” (MARA) project estimated the suitability of malaria transmission in a given map grid-square based on environmental factors [6]. In 2007, the “Malaria Atlas Project” (MAP) published the first map that estimated malaria parasite rate in most sub-Saharan countries using survey and facility-based data [2,7].

Population risk of malaria is not the only influencing factor for a household’s ownership of ITNs. Many studies over the past 15 years have examined other household-level factors that influence ITN ownership, including urban or rural residence [8-10], relative household wealth [9,11-13], and the presence of a household member who was part of a distribution campaign’s target population, chiefly pregnant women and children under five years of age [9,14,15]. These household-level factors are interrelated. Wealthier households are more likely to be in urban areas and poorer households are more likely to have children under five years of age or pregnant women because fertility is consistently higher in poorer households. Furthermore, in some countries ITN distribution activities have focused on rural areas considered more likely to be malaria prone, while some have provided free or low-cost ITNs to poor households.

The type of ITN distribution activities in a country also influences household ownership of an ITN along with the year the survey took place. Before large-scale donor assistance programs were procuring nets, many countries focused their ITN distribution activities on women during antenatal care visits and/or young children during routine vaccination campaigns. Recent studies have shown that households with a member of these target populations are more likely to own a mosquito net than households without a target population member [9]. These were the two groups with the highest morbidity and mortality due to malaria. In recent years, RBM and WHO have set goals to target all households and all individuals at risk of malaria through universal coverage campaigns. The timing, roll-out, and implementation of universal distribution campaigns vary within and across countries but universal coverage campaigns started in many countries after 2008 [16]. Additionally, many countries have maintained their ITN distribution through routine programs such as antenatal care and vaccination clinics, to ensure that pregnant women and young children are covered by nets. Table 2 shows the years for the start of distribution of free nets and distribution to all ages for the countries included in this analysis.

**Table 2:** Surveys included in analysis

**Country**	Type*	**Year**	**Field work dates**	**Months of field work**	**Total clusters**	**% of households with missing GPS**	**Total households with GPS**	**Total de facto household members with GPS**	ITNs/LLINs distributed free of charge **	ITNs/LLINs distributed to all age groups **
Angola	MIS	2011	1/2011-5/2011	4	238	3.4	7,753	35,431	2001	No
Burkina Faso	DHS	2010	4/2010-12/2010	8	573	5.6	13,617	71,354	2007	1998
Burundi	DHS	2010	8/2010-1/2011	6	376	0.0	8,596	37,702	2000	2000
Cameroon	DHS	2011	1/2011-8/2011	8	578	0.2	14,189	65,225	No	No
DRC	DHS	2007	2/2007-6/2007	4	300	2.3	8,679	42,337	2006	2008
Ghana	DHS	2008	9/2008-11/2008	3	411	1.7	11,574	40,883	2004	2010
Kenya	DHS	2008-09	11/2008-2/2009	4	398	0.3	9,033	35,772	2006	2010
Liberia	MIS	2011	9/2011-1/2012	5	150	0.0	4,162	17,255	2005	2008
Madagascar	DHS	2008-09	11/2008-8/2009	10	594	1.5	17,578	76,131	2004	2009
Malawi	MIS	2012	3/2012-4/2012	2	140	0.0	3,404	13,652	2006	2010
Mali	AMP	2010	8/2010-10/2010	3	109	2.8	1,578	8,749	2005	No
Namibia	DHS	2006-07	11/2006-3/2007	5	500	1.8	9,036	37,957	1998	No
Nigeria	MIS	2010	10/2010-12/2010	3	239	0.0	5,895	28,284	2001	2009
Rwanda	DHS	2010	9/2010-3/2011	7	492	0.0	12,540	52,877	2004	No
Senegal	DHS	2011	10/2010-4/2011	7	391	1.5	7,780	67,087	1998	1998
Sierra Leone	DHS	2008	4/2008-6/2008	3	353	0.8	7,224	38,348	2010	No
Tanzania	AIS	2011-12	12/2011-5/2012	6	583	1.7	9,862	46,044	No^	No^^
Uganda	DHS	2011	6/2011-12/2011	6	404	1.0	8,939	39,692	2006	2013
Zambia	DHS	2007	4/2007-10/2007	7	319	0.0	7,164	31,332	Yes	Yes
Zimbabwe	DHS	2010-11	9/2010-3/2011	7	406	3.2	9,442	37,475	2009	2009

*MIS = Malaria Indicator Survey, DHS = Demographic and Health Survey, AMP = Anemia and Malaria Prevalence Survey, AIS = AIDS Indicator Survey.

**Dates obtained from World Malaria Report 2013 Country Profiles [22].

^Zanzibar 2005, ^^Zanzibar 2008.

This analysis proposes an approach classifying the population at risk of malaria in nationally representative population-based survey that is not dependent on being able to identify malaria risk in a location during the survey design, but rather being able to assign risk zones after a survey has already been completed. The approach also facilitates comparison over time since risk zones can be applied to several surveys to create an estimated trend. This trend comparison may not be possible using other approaches including those proposed in the MIS sampling manual or the RBM guidelines. The population at intermediate/high risk of malaria is defined to be the population in survey clusters located in areas with greater than or equal to 5% malaria prevalence; the coverage indicators are compared for the national level and “endemicity zones”.

## Methods

### Household data

This analysis uses data from nationally representative population-based surveys including Demographic and Health Surveys (DHS), Malaria Indicator Surveys (MIS), an AIDS Indicator Survey (AIS), and an Anemia and Malaria Prevalence Survey (AMP). The surveys were primarily funded by the US Agency for International Development (USAID) through the Demographic and Health Surveys Program. Criteria for dataset inclusion in the analysis were: survey fieldwork completed between 2006 and 2012; dataset made publicly available by May 1, 2013; geographic locations of survey clusters available; and survey incorporated the mosquito net roster questions. Details about the 20 surveys included are listed in Table 2[17]. No primary data collection was done for this study, only secondary data analysis of publicly available datasets was conducted. Ethical approval for the survey data collection is outlined in each survey final report but generally was obtained from the appropriate Institutional Review Boards (IRB) in the survey country and from the ICF International IRB.

Key variables derived from datasets:

• Insecticide-treated nets (ITNs) are defined as either long-lasting insecticide-treated nets (LLINs) or conventional mosquito nets that were treated with insecticide in the last 12 months or were purchased pre-treated within the last 12 months.

• Urban–rural residence is defined in the survey sample design based on definitions established by each country.

• Wealth quintiles were created using information primarily on the ownership of household assets [18].

• The target population within the household is defined as currently pregnant women or children under five are usual (*de jure*) members of the household.

The outcome variables of interest in this analysis were two international indicators for measuring household ITN ownership (Table 1).

• Proportion of households with at least one ITN.

• Proportion of households with at least one ITN for every two *de facto* persons.

•*De facto* persons are all individuals who stayed in the household the night preceding the survey, including usual residents and visitors.

### Geographic location of clusters

The geographic locations of the centroid of survey clusters (enumeration areas or primary sampling units) are collected in most of the surveys through Global Positioning System (GPS) receivers. To maintain the confidentiality of the respondents, in accordance with IRB requirements, the GPS location for each cluster is randomly displaced. Urban clusters are displaced up to 2 kilometers, while rural clusters are displaced up to 5 kilometers with 1% of rural clusters displaced up to 10 kilometers [19]. Clusters were excluded from analysis if the GPS location is listed as missing (see the percentage of households in clusters with missing GPS locations in Table 2).

### Malaria endemicity

The analysis uses the Malaria Atlas Project (MAP) data layers from 2010 as a proxy for malaria endemicity. The MAP 2010 data estimates the *Plasmodium falciparum* parasite rate, age-standardized to 2–10 years (*Pf*PR_2–10_) in a given location. Interpolated gridded raster layers of the estimated *Pf*PR_2–10_ were created for the whole world using a Bayesian geo-statistical framework giving preference to more recent data and incorporating relevant covariates [2].

### Creation of endemicity variables

Raster values from the MAP data layers were assigned to each survey cluster’s GPS location using the “Extract Multi Values to Points” function in ArcGIS for Desktop version 10.1 with Spatial Analyst toolbox (ESRI, Redlands, CA). The raster values, converted to percentages, were classified into two zones: (1) No/Low risk (*Pf*PR_2–10_ < 5%) and (2) Intermediate/High risk (*Pf*PR_2–10_ ≥ 5%). Cluster locations outside the raster extent along the coastline were classified manually by assigning the cluster the endemicity zone of the nearest raster square. Analysis of the error associated with DHS displacement shows that for raster layers with medium to high autocorrelation such as the classified MAP data, a point extraction is an appropriate approach leading to little misclassification [20].

### Statistical analysis

Statistical analysis was done using STATA version 12 SE (STATA Corporation, College Station, TX). The “svy” suite of commands was used to account for the multi-stage sampling design, and household weights were used for all bivariate and logistic regression models.

The bivariate analysis compares the percentages of households with at least one ITN per household or at least one ITN per two persons in the national total to the percentage in the intermediate/high endemicity zone. The STATA post-estimation command “suest” (seemingly unrelated estimation) approach was used to determine statistically significant differences between non-independent samples [21]. This method uses the parameter estimates and associated variance and co-variance matrices, which make this approach appropriate even when the estimates are obtained from non-independent data (i.e., overlapping data such as national compared with specific sub-national groups). P-values of less than 0.05 were considered statistically significant for the comparison between the national total and the intermediate/high endemicity zone. The tables include 95% confidence intervals for reference. The bivariate analysis includes estimates for each country by endemicity zone and for the urban and rural locations separately.

Logistic regressions include only endemicity zones in the unadjusted model (reference: no/low endemicity) and in the adjusted models controlled for the following covariates: urban–rural residence, having a target population member in the household, wealth, and household size. An odds ratio (OR) with p-values of less than 0.05 was considered statistically significant.

## Results

**Figure 1:**
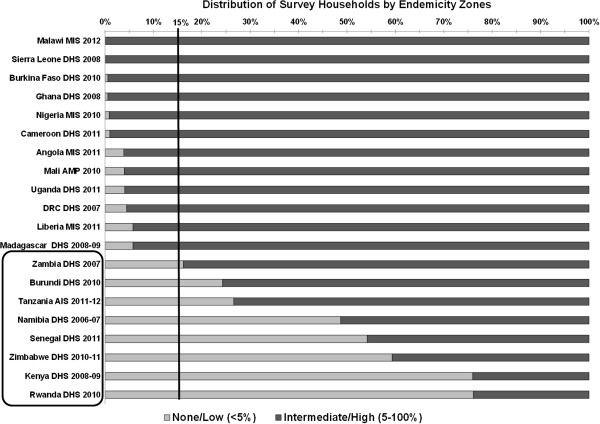
Distribution of survey households by endemicity zones.

In the majority of the countries analyzed, there is a fairly homogenous distribution of the population at risk of malaria across survey locations with the majority of households in the intermediate/high endemicity zone. However, in eight countries at least 15% of the sample households are in the no/low endemicity zone. Figure 1 illustrates the distribution of survey households by endemicity zones in the 20 countries studied.

For the purpose of this analysis, countries in which 15% or more of the population was in the no/low endemicity zone were categorized as having “heterogeneous” malaria endemicity. This cut-off was identified through data exploration and review of the results. The rest of this analysis focuses on the eight countries categorized as heterogeneous: Burundi, Kenya, Namibia, Rwanda, Senegal, Tanzania, Zambia, and Zimbabwe. The complete data tables for all 20 surveys can be found in Additional files 1, 2, 3 and 4.

### Ownership of at least one ITN per household

**Figure 2:**
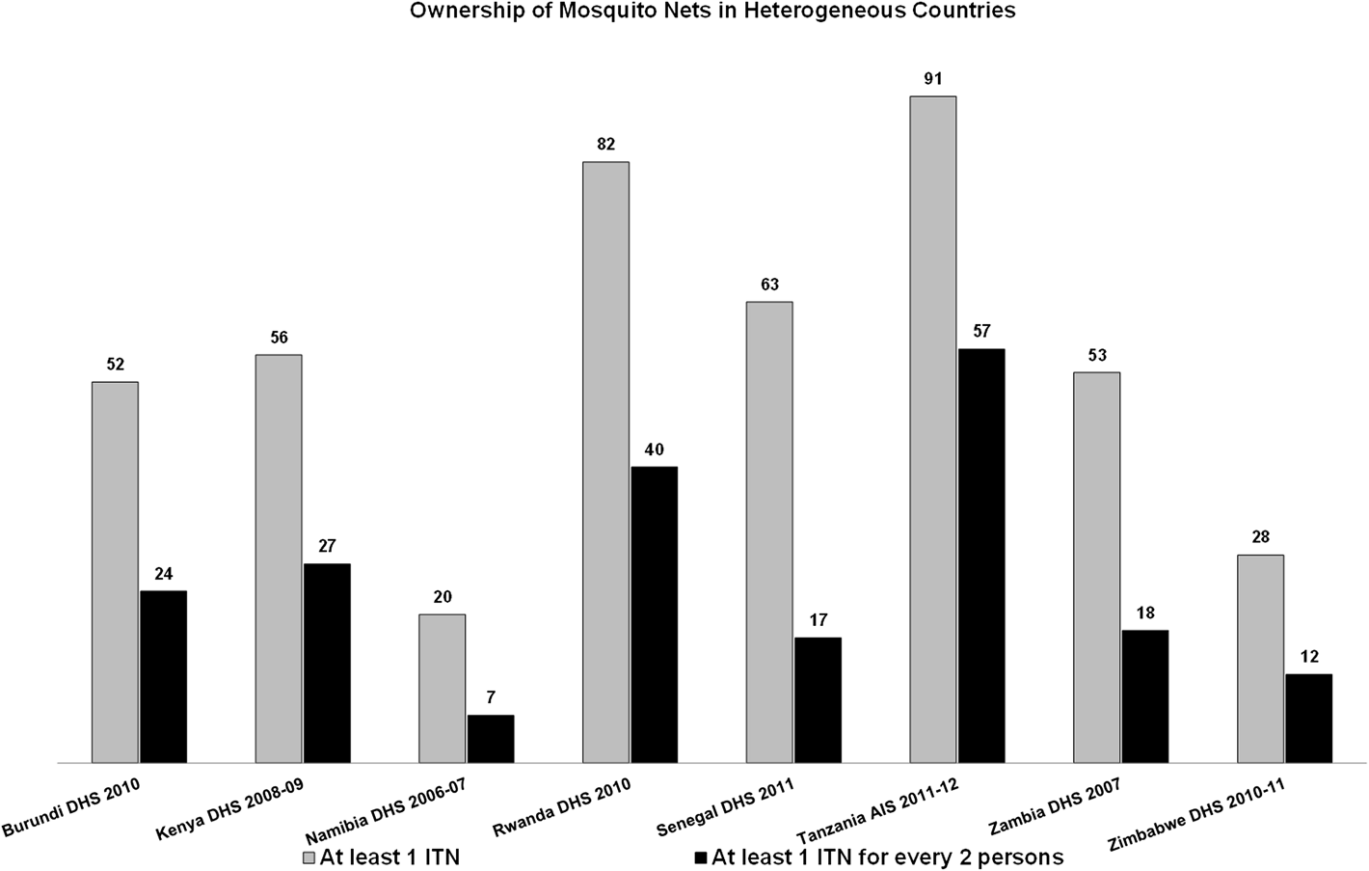
Ownership of mosquito nets in heterogeneous countries.

Ownership of at least one ITN per household varies widely among the countries with heterogeneous malaria zones, ranging from 20% in Namibia to 91% in Tanzania (Figure 2). It should be noted that the Namibia survey is from 2006 when ITNs and specifically LLINs were not as widely distributed as they were in 2011–2012 when the Tanzania survey was conducted.

Examining household ownership of at least one ITN by endemicity zones shows that in all of the heterogeneous endemicity countries, households in the intermediate/high endemicity zones are significantly more likely to own at least one ITN than all households together (Table 3). For example, in Zimbabwe the national ownership of ITNs is 28%, but ownership in the intermediate/high endemicity zone is 46%.

**Table 3:** Percentage of households in heterogeneous countries that own at least one ITN by residence and malaria endemicity zone

		**Endemicity**				**National total**			
		**None/low (< 5%)**		**Intermediate/high (5-100%)**					
**Country/Survey/Year**	**Residence**	**Estimate**	**95% CI**	**Estimate**	**95% CI**	**Estimate**	**95% CI**	**N**	**Intermediate/high significantly different from total**
Burundi DHS 2010	National	39.9	(32.5, 47.3)	55.9	(52.4, 59.4)	52.0	(48.8, 55.2)	8,596	*
Burundi DHS 2010	Urban	40.1	(32.5, 47.7)	54.1	(50.2, 57.9)	50.4	(46.9, 54.0)	7,817	
Burundi DHS 2010	Rural	34.9	(20.3, 49.6)	70.7	(65.8, 75.5)	67.7	(62.6, 72.8)	779	*
Kenya DHS 2008-09	National	51.1	(47.4, 54.8)	70.1	(66.3, 74.0)	55.7	(52.7, 58.7)	9,033	*
Kenya DHS 2008-09	Urban	49.5	(45.0, 54.0)	68.9	(64.8, 73.1)	54.9	(51.5, 58.3)	6,682	*
Kenya DHS 2008-09	Rural	54.9	(47.9, 61.8)	77.6	(69.8, 85.4)	57.8	(51.1, 64.5)	2,350	*
Namibia DHS 2006-07	National	9.3	(7.5, 11.0)	30.7	(28.7, 32.8)	20.3	(19.0, 21.6)	9,031	*
Namibia DHS 2006-07	Urban	17.3	(12.8, 21.8)	32.0	(29.5, 34.4)	28.7	(26.7, 30.7)	4,870	*
Namibia DHS 2006-07	Rural	6.6	(5.2, 8.0)	25.3	(22.0, 28.6)	10.4	(9.1, 11.7)	4,161	*
Rwanda DHS 2010	National	79.4	(78.2, 80.6)	90.2	(88.6, 91.8)	82.0	(81.0, 82.9)	12,540	*
Rwanda DHS 2010	Urban	78.5	(77.0, 79.9)	90.2	(88.5, 91.9)	81.6	(80.5, 82.7)	10,781	*
Rwanda DHS 2010	Rural	84.0	(81.6, 86.5)	89.6	(85.0, 94.2)	84.5	(82.2, 86.7)	1,759	*
Senegal DHS 2011	National	51.1	(46.1, 56.0)	76.8	(73.9, 79.8)	62.9	(59.7, 66.0)	7,793	*
Senegal DHS 2011	Urban	67.6	(61.6, 73.5)	76.0	(72.6, 79.3)	73.4	(70.5, 76.3)	3,929	*
Senegal DHS 2011	Rural	44.5	(38.1, 50.8)	79.5	(73.1, 85.9)	52.2	(46.9, 57.4)	3,864	*
Tanzania AIS 2011-12	National	88.0	(86.1, 89.9)	91.9	(90.9, 92.9)	90.9	(90.0, 91.8)	9,882	*
Tanzania AIS 2011-12	Urban	90.0	(87.7, 92.4)	93.1	(92.1, 94.2)	92.4	(91.4, 93.3)	7,322	
Tanzania AIS 2011-12	Rural	83.3	(79.5, 87.1)	88.2	(85.4, 91.0)	86.7	(84.5, 88.9)	2,560	*
Zambia DHS 2007	National	48.4	(43.6, 53.3)	54.3	(51.5, 57.1)	53.3	(50.8, 55.8)	7,164	*
Zambia DHS 2007	Urban	46.9	(32.0, 61.8)	53.9	(50.5, 57.3)	53.7	(50.4, 57.1)	4,685	
Zambia DHS 2007	Rural	48.6	(43.4, 53.7)	55.5	(51.0, 60.1)	52.6	(49.2, 55.9)	2,479	
Zimbabwe DHS 2010-11	National	16.1	(14.2, 18.1)	46.2	(40.9, 51.5)	28.4	(25.6, 31.2)	9,467	*
Zimbabwe DHS 2010-11	Urban	12.0	(8.7, 15.3)	46.6	(40.9, 52.4)	31.4	(27.3, 35.5)	6,249	*
Zimbabwe DHS 2010-11	Rural	20.1	(18.1, 22.1)	42.0	(31.1, 53.0)	22.5	(20.2, 24.7)	3217	*

*Indicates Intermediate/high estimates that are significantly different from the national total by row (p-value < 0.05).

Table 4 shows the adjusted and unadjusted odds ratios of ownership of one ITN per household for the heterogeneous countries. Results for all models and all countries can be found in Additional file 2. In all heterogeneous endemicity countries, even after controlling for all other factors, households in the intermediate/high endemicity zone had significantly higher odds of owning at least one ITN than households in the no/low endemicity zone. The ORs for the intermediate/high endemicity zone are fairly consistent in the adjusted and unadjusted models. In Senegal, for example, the unadjusted odds of having one ITN per household was 3.17 times higher in intermediate/high endemicity zones than in no/low endemicity zones, and the odds ratio was reduced to 2.10 after controlling for other covariates. Of note, having a member of the target population in the household was a significant predictor of owning at least one ITN in every heterogeneous country, with odds ratios ranging from 1.19 to 4.30.

**Table 4:** Logistic regressions of ownership of at least one ITN in heterogeneous countries

**Countries/Factors**	**Unadjusted**	**Adjusted**	**Countries/Factors**	**Unadjusted**	**Adjusted**
	**OR (95% CI)**	**OR (95% CI)**		**OR (95% CI)**	**OR (95% CI)**
**Burundi DHS 2010**			**Senegal DHS 2011**		
Intermediate/high endemicity	1.90* (1.35,2.68)	1.91* (1.32,2.77)	Intermediate/high endemicity	3.17* (2.46,4.10)	2.10* (1.66,2.67)
Target population in household		1.86* (1.63,2.11)	Target population in household		1.63* (1.41,1.90)
Rural household		1.25 (0.92,1.72)	Rural household		0.88 (0.66,1.16)
Second wealth quintile		1.67* (1.41,1.97)	Second wealth quintile		1.11 (0.86,1.44)
Middle wealth quintile		1.93* (1.58,2.35)	Middle wealth quintile		1.08 (0.79,1.49)
Fourth wealth quintile		2.31* (1.88,2.85)	Fourth wealth quintile		0.68* (0.49,0.94)
Highest wealth quintile		2.80* (2.20,3.56)	Highest wealth quintile		0.48* (0.33,0.70)
**Kenya DHS 2008-09**			**Tanzania AIS 2011-12**		
Intermediate/high endemicity	2.25* (1.77,2.85)	2.31* (1.80,2.96)	Intermediate/high endemicity	1.55* (1.22,1.97)	1.48* (1.17,1.86)
Target population in household		2.20* (1.83,2.64)	Target population in household		1.59* (1.26,1.99)
Rural household		1.28 (0.91,1.79)	Rural household		0.68 (0.46,1.03)
Second wealth quintile		1.52* (1.25,1.84)	Second wealth quintile		1.26 (0.94,1.69)
Middle wealth quintile		1.85* (1.41,2.43)	Middle wealth quintile		2.10* (1.56,2.82)
Fourth wealth quintile		1.57* (1.15,2.14)	Fourth wealth quintile		1.67* (1.20,2.34)
Highest wealth quintile		1.62* (1.14,2.30)	Highest wealth quintile		1.01 (0.66,1.55)
**Namibia DHS 2006-07**			**Zambia DHS 2007**		
Intermediate/high endemicity	4.36* (3.45,5.49)	3.09* (2.32,4.12)	Intermediate/high endemicity	1.26* (1.01,1.58)	1.51* (1.19,1.93)
Target population in household		1.70* (1.48,1.96)	Target population in household		1.42* (1.26,1.60)
Rural household		0.56* (0.44,0.72)	Rural household		0.60* (0.45,0.81)
Second wealth quintile		1.35* (1.11,1.64)	Second wealth quintile		1.31* (1.06,1.62)
Middle wealth quintile		1.38* (1.11,1.72)	Middle wealth quintile		1.48* (1.19,1.84)
Fourth wealth quintile		1.40* (1.05,1.86)	Fourth wealth quintile		2.03* (1.50,2.75)
Highest wealth quintile		0.97 (0.67,1.41)	Highest wealth quintile		3.53* (2.41,5.17)
**Rwanda DHS 2010**			**Zimbabwe DHS 2010-11**		
Intermediate/high endemicity	2.38* (1.95,2.90)	2.38* (1.92,2.95)	Intermediate/high endemicity	4.47* (3.46,5.77)	5.53* (4.10,7.46)
Target population in household		4.30* (3.58,5.17)	Target population in household		1.19* (1.03,1.38)
Rural household		1.08 (0.86,1.35)	Rural household		1.13 (0.85,1.51)
Second wealth quintile		1.40* (1.22,1.61)	Second wealth quintile		0.94 (0.76,1.15)
Middle wealth quintile		2.03* (1.70,2.43)	Middle wealth quintile		1.04 (0.83,1.30)
Fourth wealth quintile		2.67* (2.24,3.18)	Fourth wealth quintile		1.18 (0.91,1.53)
Highest wealth quintile		2.93* (2.39,3.58)	Highest wealth quintile		1.67* (1.20,2.31)

OR = Odds ratio, CI = Confidence interval, *p < 0.05.

Reference Categories: Intermediate/high endemicity (Ref: No\low endemicity), Target population in household (Ref: No member of target population in household), Rural household (Ref: Urban household), Wealth (second, middle, fourth, highest quintile) (Ref: Lowest).

### Ownership of at least one ITN for every two persons

Ownership of at least one ITN for every two persons in the household varies across the eight heterogeneous countries, but is considerably lower than the percentage of households owning at least one ITN (Figure 2). Differences in ITN ownership between the national and intermediate/high endemicity zone estimates were significant in Burundi, Kenya, Namibia, Rwanda, Senegal, and Zimbabwe, but not in Tanzania and Zambia (Table 5).

**Table 5:** Percentage of households in heterogeneous countries that own at least one ITN for every two de facto persons in the household by residence and malaria endemicity zone

		**Endemicity**				**National total**			
		**None/low (< 5%)**		**Intermediate/high (5-100%)**					
**Country/Survey/Year**	**Residence**	**Estimate**	**95% CI**	**Estimate**	**95% CI**	**Estimate**	**95% CI**	**N**	**Intermediate/high significantly different from total**
Burundi DHS 2010	National	16.6	(11.2, 22.0)	25.7	(23.1, 28.3)	23.5	(21.1, 25.8)	8,596	*
Burundi DHS 2010	Urban	16.9	(11.3, 22.4)	24.9	(22.0, 27.7)	22.8	(20.3, 25.3)	7,817	
Burundi DHS 2010	Rural	6.7	(2.6, 10.8)	32.5	(28.3, 36.7)	30.4	(26.2, 34.6)	779	*
Kenya DHS 2008-09	National	25.7	(23.3, 28.1)	31.9	(27.3, 36.5)	27.2	(25.0, 29.3)	9,033	*
Kenya DHS 2008-09	Urban	21.2	(18.2, 24.2)	28.3	(23.9, 32.8)	23.2	(20.8, 25.7)	6,682	
Kenya DHS 2008-09	Rural	36.2	(31.9,40.4)	54.1	(40.7, 67.5)	38.4	(33.9, 43.0)	2,350	*
Namibia DHS 2006-07	National	3.7	(2.8, 4.6)	9.1	(7.9, 10.3)	6.5	(5.7, 7.2)	9,031	*
Namibia DHS 2006-07	Urban	5.9	(3.2, 8.7)	9.0	(7.6, 10.3)	8.3	(7.2, 9.4)	4,870	
Namibia DHS 2006-07	Rural	2.9	(2.2, 3.7)	9.6	(7.3, 12.0)	4.3	(3.6, 5.1)	4,161	*
Rwanda DHS 2010	National	37.9	(36.3, 39.5)	48.1	(45.4, 50.8)	40.4	(39.0, 41.7)	12,540	*
Rwanda DHS 2010	Urban	35.3	(33.4, 37.2)	47.8	(45.0, 50.6)	38.6	(37.1, 40.1)	10,781	*
Rwanda DHS 2010	Rural	50.8	(46.6, 55.0)	54.5	(43.2, 65.8)	51.1	(47.1, 55.0)	1,759	*
Senegal DHS 2011	National	10.9	(9.0,1 2.8)	24.6	(22.6, 26.5)	17.1	(15.7, 18.5)	7,793	*
Senegal DHS 2011	Urban	13.6	(10.2, 17.1)	23.6	(21.3, 26.0)	20.6	(18.7, 22.4)	3,929	*
Senegal DHS 2011	Rural	9.8	(7.6,1 2.0)	27.5	(24.2, 30.7)	13.7	(11.7, 15.7)	3,864	*
Tanzania AIS 2011-12	National	54.5	(51.3, 57.7)	57.2	(55.3, 59.1)	56.5	(54.9, 58.1)	9,882	
Tanzania AIS 2011-12	Urban	52.4	(48.4, 56.4)	55.0	(52.8, 57.2)	54.4	(52.4, 56.3)	7,322	
Tanzania AIS 2011-12	Rural	59.4	(54.1, 64.6)	64.1	(60.8, 67.3)	62.6	(59.9, 65.4)	2,560	*
Zambia DHS 2007	National	16.6	(13.0, 20.1)	18.4	(16.6, 20.1)	18.1	(16.5, 19.7)	7,164	
Zambia DHS 2007	Urban	19.1	(8.7,2 9.5)	17.8	(15.7, 19.8)	17.8	(15.8, 19.8)	4,685	
Zambia DHS 2007	Rural	16.3	(12.5, 20.1)	20.3	(16.6, 24.0)	18.6	(16.0, 21.2)	2,479	
Zimbabwe DHS 2010-11	National	5.7	(4.8, 6.5)	21.4	(17.6, 25.3)	12.1	(10.3, 13.9)	9,467	*
Zimbabwe DHS 2010-11	Urban	3.6	(2.4, 4.9)	21.4	(17.2, 25.5)	13.6	(11.0, 16.2)	6,249	*
Zimbabwe DHS 2010-11	Rural	7.6	(6.5, 8.7)	22.2	(14.2, 30.1)	9.2	(7.8, 10.6)	3217	*

*Indicates intermediate/high estimates that are significantly different from the national total by row (p-value < 0.05).

In the logistic regression models, residing in an intermediate/high endemicity zone was a significant predictor of households owning at least one ITN for every two persons in the household (Table 6 and Additional file 4 for all models and all countries). This result was consistent for all heterogeneous transmission countries. When household size was included in the regression (adjusted model), having a member of the target population in the household was no longer a significant predictor in most countries, but having a larger number of persons in the household was a significant negative predictor of owning at least one ITN for every two persons in every heterogeneous country. Further investigation showed that households with pregnant women or children under five are more likely to be larger households than households without individuals from the target population.

**Table 6:** Logistic regressions of ownership at least one ITN for every two de facto persons in household for heterogeneous countries

	**Unadjusted**	**Adjusted**		**Unadjusted**	**Adjusted**
**Country/Factors**	**OR (95% CI)**	**OR (95% CI)**	**Countries/Factors**	**OR (95% CI)**	**OR (95% CI)**
**Burundi DHS 2010**			**Senegal DHS 2011**		
Intermediate/high endemicity	1.74* (1.15,2.64)	1.73* (1.11,2.71)	Intermediate/high endemicity	2.67* (2.14,3.33)	3.09* (2.40,3.97)
Target population in household		1.07 (0.93,1.23)	Target population in household		0.9 (0.75,1.07)
Rural household		1.1 (0.76,1.60)	Rural household		0.79 (0.60,1.05)
Second wealth quintile		1.50* (1.20,1.88)	Second wealth quintile		1.1 (0.88,1.38)
Middle wealth quintile		1.81* (1.45,2.26)	Middle wealth quintile		1.28 (0.95,1.72)
Fourth wealth quintile		1.89* (1.49,2.39)	Fourth wealth quintile		0.9 (0.65,1.25)
Highest wealth quintile		2.36* (1.75,3.18)	Highest wealth quintile		0.69 (0.46,1.03)
Total number of individuals in household (de facto)		0.69* (0.67,0.71)	Total number of individuals in household (de facto)		0.79* (0.77,0.81)
**Kenya DHS 2008-09**			**Tanzania AIS 2011-12**		
Intermediate/high endemicity	1.35* (1.05,1.74)	2.03* (1.56,2.64)	Intermediate/high endemicity	1.12 (0.96,1.30)	1.39* (1.20,1.63)
Target population in household		0.88 (0.71,1.08)	Target population in household		0.62* (0.54,0.71)
Rural household		1.12 (0.78,1.60)	Rural household		0.81 (0.64,1.02)
Second wealth quintile		1.71* (1.33,2.19)	Second wealth quintile		1.17 (0.97,1.40)
Middle wealth quintile		2.55* (1.90,3.42)	Middle wealth quintile		1.72* (1.45,2.05)
Fourth wealth quintile		2.85* (2.06,3.96)	Fourth wealth quintile		2.06* (1.70,2.50)
Highest wealth quintile		3.49* (2.29,5.31)	Highest wealth quintile		1.79* (1.39,2.30)
Total number of individuals in household (de facto)		0.79* (0.76,0.82)	Total number of individuals in household (de facto)		0.74* (0.72,0.76)
**Namibia DHS 2006-07**			**Zambia DHS 2007**		
Intermediate/high endemicity	2.61* (1.92,3.55)	2.92* (1.95,4.38)	Intermediate/high endemicity	1.13 (0.85,1.51)	1.77* (1.22,2.55)
Target population in household		1.12 (0.78,1.61)	Target population in household		0.9 (0.75,1.08)
Rural household		0.61* (0.43,0.86)	Rural household		0.57* (0.40,0.80)
Second wealth quintile		1.31 (0.93,1.83)	Second wealth quintile		1.3 (0.94,1.81)
Middle wealth quintile		1.46* (1.05,2.02)	Middle wealth quintile		1.52* (1.09,2.13)
Fourth wealth quintile		1.88* (1.25,2.84)	Fourth wealth quintile		2.39* (1.62,3.52)
Highest wealth quintile		1.2 (0.73,1.98)	Highest wealth quintile		5.47* (3.40,8.81)
Total number of individuals in household (de facto)		0.73* (0.69,0.76)	Total number of individuals in household (de facto)		0.64* (0.61,0.67)
**Rwanda DHS 2010**			**Zimbabwe DHS 2010-11**		
Intermediate/high endemicity	1.52* (1.33,1.73)	1.68* (1.44,1.95)	Intermediate/high endemicity	4.54* (3.42,6.02)	6.79* (5.09,9.06)
Target population in household		1.29* (1.15,1.43)	Target population in household		0.96 (0.72,1.27)
Rural household		1.07 (0.86,1.34)	Rural household		1.04 (0.75,1.44)
Second wealth quintile		1.40* (1.23,1.59)	Second wealth quintile		0.91 (0.70,1.19)
Middle wealth quintile		1.95* (1.69,2.25)	Middle wealth quintile		0.98 (0.74,1.30)
Fourth wealth quintile		2.25* (1.94,2.61)	Fourth wealth quintile		1.1 (0.79,1.54)
Highest wealth quintile		3.99* (3.29,4.83)	Highest wealth quintile		1.78* (1.20,2.63)
Total number of individuals in household (de facto)		0.64* (0.63,0.66)	Total number of individuals in household (de facto)		0.70* (0.66,0.73)

OR = Odds ratio, CI = Confidence interval, *p < 0.05.

Reference Categories: Intermediate/high endemicity (Ref: No\low endemicity), Target population in household (Ref: No member of target population in household), Rural household (Ref: Urban household), Wealth (second, middle, fourth, highest quintile) (Ref: Lowest), Total number of individuals in household (continuous).

## Discussion

This analysis demonstrates that the population at risk of malaria is a crucial factor to take into account when examining intervention coverage estimates of ITN ownership in countries with heterogeneous malaria endemicity (Table 1). Countries with at least 15% of their surveyed households living in the no/low endemicity zone were classified as having heterogeneous malaria endemicity (Figure 1). Using a cutoff point as low as 10% would not have changed the eight countries considered to have heterogeneous endemicity.

ITN ownership estimates in most heterogeneous countries were significantly higher in the intermediate/high endemicity zone than in the country as a whole. This pattern is found in both the bivariate and logistic regression analyses of household ownership of at least one ITN. Although household ownership of at least one ITN is fairly high in many countries, both heterogeneous and homogenous, the percentage of households meeting the target of at least one ITN for every two persons in the household is much lower. This finding highlights the difference between what the two coverage indicators are measuring. The indicator of at least one ITN per household measures the reach of ITNs and may reflect the legacy of distribution activities that focused on households with pregnant women and young children. The indicator of at least one ITN for every two persons in the household shows the gap in achieving universal household coverage and is highly dependent on the size of the household. The data show that this gap is smaller in zones with higher endemicity than in other zones, which could be linked to universal distribution campaigns targeting areas of higher risk.

Along with understanding the risk of malaria in the survey locations, understanding a country’s ITN distribution campaign history is important in interpreting the intervention coverage estimates. The surveys included in this analysis were conducted between 2006 and 2011, in many cases during periods right on the cusp of the implementation of free and universal distribution campaigns in those countries. For that reason, some surveys may not have recorded changes in ownership linked to these distribution activities.

Limitations of this analytical approach relate to data selection and geographic uncertainty. Consideration of the malaria endemicity data layer to use remains especially important as endemicity changes over time and would be expected to decrease as interventions increased. This analysis used the MAP 2010 data layer because it included data that was closest in time to the majority of the surveys included in the analysis. The GPS data on the survey locations have some inherent uncertainties due to their displacement and the point extraction used. These effects are estimated to be relatively small due to the crudeness of the endemicity zones used and the uncertainties in the MAP data themselves. This uncertainty likely has little effect on the conclusions drawn.

## Conclusion

Coverage estimates of ITNs are generally calculated at the national level using population-based surveys, but they are intended to be a proxy for coverage among the population at risk of malaria. For surveys in countries where malaria is endemic and the entire population is at risk, national coverage rates provide good estimates of coverage among the population at risk regardless of the survey sampling or identification of malaria zones. In countries with substantial variation in malaria endemicity, however, some zones of the country may be at no or low risk of malaria (due to geographic or climate factors) and, therefore, ITN coverage would be expected to be low in those areas. The inclusion of these no- or low-risk areas in national-level estimates can bias coverage estimates downward, making national coverage rates calculated from those surveys poor proxies of coverage among the population at risk of malaria. Such underestimates make it difficult for countries to track their progress in achieving universal ITN coverage, especially when examining results from surveys not designed to identify areas of malaria risk.

Of note, as malaria intervention coverage continues to increase and the parasitemia burden (as calculated by *Pf*PR_2–10_) in the population decreases, the actual risk of being infected by the parasite may not decrease at the same pace. At the same time, ITN are used as part of a larger vector control strategy which should be maintained for a period of time to avoid rebound, even as parasitemia decreases in the region. High ITN coverage would need to be maintained for some time in the zones that previously had higher risk of malaria. This makes understanding the historical burden of malaria in a location important, as well as recognizing that changes in climate and other environmental factors might bring increased risk of malaria to zones that were previously at low risk. This sustained high ITN coverage can be seen in countries such as Rwanda, Senegal, and Tanzania whose malaria control efforts have succeeded in shifting some areas that were previously in intermediate/high endemicity zones to now be no/low endemicity zones. In contrast, most of the no/low malaria zones in Kenya, Namibia, and Zimbabwe were never at high risk of malaria, and malaria control efforts did not usually take place in those areas. Local knowledge about endemicity zones and historical malaria transmission patterns should be considered when using a geographic classification method to identify populations at risk of malaria. Although this study only examined two standard international indicators related to ownership of insecticide-treated mosquito nets, it should be noted that endemicity zones likely influence all prevention intervention indicators that are reported at a national level, such as use of ITNs, intermittent preventive treatment for malaria during pregnancy (IPTp), and malaria diagnosis and treatment.

Results from this study indicate that national coverage estimates are generally biased downwards in countries where more than 10-15% of the population lives in endemicity zones with less than 5% malaria parasitemia. Using the approach presented in this analysis countries with this type of heterogeneity can obtain more accurate estimates of ITN coverage among the population at risk of malaria, from surveys not otherwise designed to identify this population. The proposed approach uses the GPS locations of the survey clusters to assign endemicity zones using the Malaria Atlas Project data layers, allows for identification of malaria risk in a location after a survey has already been completed. Incorporating this basic and easily reproducible approach into creating estimates of ITN coverage is applicable, and even preferable, in countries with populated areas at no/low risk of malaria. The extension of this type of analysis to other malaria interventions can provide further valuable information to measure trends in intervention coverage estimates and support evidence-based decision-making. This approach, as part of a strategic planning process, can help ensure that the highest-quality information is available to inform programmatic decisions tailored for the needs of particular areas within a country.

## Abbreviations

AIS: AIDS indicator survey; AMP: Anemia and Malaria Prevalence survey; CI: Confidence interval; DHS: Demographic and Health Survey; GMAP: Global malaria action plan; GPS: Global Positioning System; HMIS: Health management information system; IPTp: Intermittent preventive treatment for malaria during pregnancy; IRB: Institutional Review Board; ITN: Insecticide-treated net; LLIN: Long-lasting insecticide-treated net; MARA: Mapping malaria risk in Africa; MAP: Malaria Atlas Project; MIS: Malaria indicator survey; OR: Odds ratio; *Pf*PR_2–10_: Plasmodium falciparum parasite rate age-standardized to 2–10 years; RBM: Roll back malaria; USAID: US Agency for International Development; WHO: World Health Organization.

## Competing interests

The authors declare that they have no competing interests.

## Authors’ contributions

CRB, SEKB, FA, and EE were equally involved in the conception and design of this project. CRB and SEKB were responsible for data acquisition and all analyses. All authors were responsible for interpretation of data. CRB drafted the manuscript and SEKB, FA, and EE critically reviewed and contributed important intellectual content. All authors read and approved the final manuscript.

## Authors’ information

CRB, SEKB, and FA are employees of ICF International, working primarily on the USAID-funded DHS Program. The DHS Program is responsible for the data collection of the survey data used in this analysis. Funding for this analysis came from USAID through The DHS Program and MEASURE Evaluation project. EE is employed by USAID, President’s Malaria Initiative. FA and EE are members of the RBM MERG.

## Supplementary Material

Additional file 1 **Percentage of households in that own at least 1 ITN by residence and by malarial endemicity zones.** Description: The data provided is the same as Table 3 in the manuscript on the percent ownership of at least one ITN but includes data from all countries included in the analysis. Click here for file

Additional file 2 **Logistic regressions of ownership of at least one ITN.** Description: The data provided is the same as Table 4 in the manuscript on the logistic regression of ownership of at least one ITN but includes data from all countries included in the analysis. Click here for file

Additional file 3 **Percentage of households that own at least one ITN for every two de facto persons in the household by residence and malaria endemicity zone.** Description: The data provided is the same as Table 5 in the manuscript on the percent of households with one ITN for every two persons in the household but includes data from all countries included in the analysis. Click here for file

Additional file 4 **Logistic Regression of Ownership of at least One ITN for every two de facto persons.** Description: The data provided is the same as Table 6 in the manuscript on the logistic regression of households with one ITN for every two persons but includes data from all countries included in the analysis. Click here for file

## References

[B1] Roll Back Malaria Partnership The Global Malari Aciton Plan: For a Malaria-Free World 2008 Geneva, Switzerland: Roll Back Malaria Partnership

[B2] Gething PW Patil AP Smith DL Guerra CA Elyazar IR Johnston GL Tatem AJ Hay SI A new world malaria map: *Plasmodium falciparum* endemicity in 2010 Malar J 2011 10 378 22185615 10.1186/1475-2875-10-378 PMC3274487

[B3] Roll Back Malaria Partnership Household Survey Indicators for Malaria Control 2013 Calverton, MD: Roll Back Malaria, MEASURE Evaluation, USAID, UNICEF, World Health Organization, CDC

[B4] MEASURE DHS Malaria Indicator Survey: Guidelines for Sampling for the Malaria Indicator Survey 2013 Calverton, Maryland: ICF International

[B5] WHO World Malaria Report: 2010 2010 Geneva Switzerland: World Health Organization

[B6] Adjuik M Bagayoko M Binka F Coetzee M Cox J Craig M Deichman U de Savigny D Fondjo E Fraser C Gouws E Kleinschmidt I Lemardeley P Lengeler C LeSueur D Omumbo J Snow R Sharp B Tnaser F Teuscher T Touré Y Towards an Atlas of Malaria Risk in Africa: First Technical Report of the MARA/ARMA Collaboration 1998 Durban, South Africa: IDRC, South African Medical Research Council, Wellcome Trust UK

[B7] Hay SI Guerra CA Gething PW Patil AP Tatem AJ Noor AM Kabaria CW Manh BH Elyazar IRF Brooker S Smith DL Moyeed RA Snow RW A world malaria map: *Plasmodium falciparum* endemicity in 2007 PLoS Med 2009 6 e1000048 19323591 10.1371/journal.pmed.1000048 PMC2659708

[B8] Noor AM Alegana VA Patil AP Snow RW Predicting the unmet need for biologically targeted coverage of insecticide-treated nets in Kenya Am J Trop Med Hyg 2010 83 854 860 20889879 10.4269/ajtmh.2010.10-0331 PMC2946756

[B9] Oresanya O Hoshen M Sofola O Utilization of insecticide-treated nets by under-five children in Nigeria: assessing progress towards the Abuja targets Malar J 2008 7 145 18667077 10.1186/1475-2875-7-145 PMC2543041

[B10] Kazembe LN Appleton CC Kleinschmidt I Geographical disparities in core population coverage indicators for roll back malaria in Malawi Int J Equity Health 2007 6 5 17610730 10.1186/1475-9276-6-5 PMC1934906

[B11] Wiseman V Scott A McElroy B Conteh L Stevens W Determinants of bed net use in the Gambia: implications for malaria control Am J Trop Med Hyg 2007 76 830 836 17488900

[B12] Ye Y Patton E Kilian A Dovey S Eckert E Can universal insecticide-treated net campaigns achieve equity in coverage and use? the case of northern Nigeria Malar J 2012 11 32 22297189 10.1186/1475-2875-11-32 PMC3312823

[B13] Barat LM Palmer N Basu S Worrall E Hanson K Mills A Do malaria control interventions reach the poor? A view through the equity lens Am J Trop Med Hyg 2004 71 174 178 15331835

[B14] Noor AM Amin AA Akhwale WS Snow RW Increasing coverage and decreasing inequity in insecticide-treated bed net use among rural Kenyan children PLoS Med 2007 4 e255 17713981 10.1371/journal.pmed.0040255 PMC1949846

[B15] Monasch R Reinisch A Steketee RW Korenromp EL Alnwick D Bergevin Y Child coverage with mosquito nets and malaria treatment from population-based surveys in african countries: a baseline for monitoring progress in roll back malaria Am J Trop Med Hyg 2004 71 232 238 15331842

[B16] Malaria Partnership Launches “Cover The Bed Net Gap” Initiative To Protect Everyone At Risk Of Malaria In Africa[http://www.rbm.who.int/globaladvocacy/pr2008-04-25b.html]

[B17] International ICF Demographic and Health Surveys (various) ICF International ed 2006-2012 Calverton, Maryland: ICF International

[B18] Rutstein SO Johnson K The DHS Wealth Index DHS Comparative Report, vol. 6 2004 Calverton, Maryland: Macro International

[B19] Burgert CR Colston J Roy T Zachary B Household Survey Cluster Displacement procedure for the Demogrpahic and Health Surveys DHS Spatial Analysis Reports, vol. 7 2013 Calverton, Maryland: ICF International

[B20] Perez-Heyrich C Warren JL Burgert CR Emch ME Guidelines on the use of DHS GPS data DHS Spatial Analysis Reports, vol. 8 2013 ICF Interactional: Calverton, Maryland

[B21] STATA STATA Base Reference Manual Q-Z Release 11 2009 College Station, Texas: STATACorp

[B22] World Health Organization World Malaria Report: 2013, Country Profiles 2013 Geneva, Switzerland: World Health Organization

